# Screening for Developmental Neurotoxicity Using PC12 Cells: Comparisons of Organophosphates with a Carbamate, an Organochlorine, and Divalent Nickel

**DOI:** 10.1289/ehp.9527

**Published:** 2006-09-06

**Authors:** Theodore A. Slotkin, Emiko A. MacKillop, Ian T. Ryde, Charlotte A. Tate, Frederic J. Seidler

**Affiliations:** Department of Pharmacology & Cancer Biology, Duke University Medical Center, Durham, North Carolina, USA

**Keywords:** acetylcholine systems, carbamates, catecholamine systems, chlorpyrifos, developmental neurotoxicity, diazinon, dieldrin, nickel, organochlorines, organophosphates

## Abstract

**Background:**

In light of the large number of chemicals that are potential developmental neurotoxicants, there is a need to develop rapid screening techniques.

**Objectives:**

We exposed undifferentiated and differentiating neuronotypic PC12 cells to different organophosphates (chlorpyrifos, diazinon, parathion), a carbamate (physostigmine), an organochlorine (dieldrin), and a metal (divalent nickel; Ni^2+^) and examined indices of cell replication and differentiation for both short- and long-term exposures.

**Results:**

In undifferentiated cells, all the agents inhibited DNA synthesis, with the greatest effect for diazinon, but physostigmine eventually produced the largest deficits in the total number of cells after prolonged exposure. The onset of differentiation intensified the adverse effects on DNA synthesis and changed the rank order in keeping with a shift away from noncholinergic mechanisms and toward cholinergic mechanisms. Differentiation also worsened the effects of each agent on cell number after prolonged exposure, whereas cell growth was not suppressed, nor were there any effects on viability as assessed with trypan blue. Nevertheless, differentiating cells displayed signs of oxidative stress from all of the test compounds except Ni^2+^, as evidenced by measurements of lipid peroxidation. Finally, all of the toxicants shifted the transmitter fate of the cells away from the cholinergic phenotype and toward the catecholaminergic phenotype.

**Conclusions:**

These studies point out the feasibility of developing cell-based screening methods that enable the detection of multiple end points that may relate to mechanisms associated with developmental neurotoxicity, revealing some common targets for disparate agents.

Recent evidence points to important contributions of exposure to environmental neurotoxicant chemicals in the marked increase in neurodevelopmental disorders, including learning disabilities, attention deficit and hyperactivity disorder, and autism spectrum disorders (Szpir 2006). Despite the increasing recognition of the importance of evaluating developmental neurotoxicity in safety assessment (Claudio et al. 2000; Eriksson 1997; Tilson 1995, 2000), the fact remains that, of > 70,000 commercial chemicals in current use, neurotoxicity of any kind has been evaluated in < 10% (Landrigan et al. 1994), and obviously, developmental neurotoxicity in a substantially smaller fraction. Even now, of the 2,000–3,000 new chemicals released each year, two-thirds never get tested for neurotoxicity, let alone developmental effects (Claudio et al. 2000), whereas between 25–40% will eventually prove to be neurotoxic (Boyes 2001). Among the many potential developmental neurotoxicants, the greatest attention has been paid to pesticides, in light of their widespread use in the home and in agriculture [U.S. Environmental Protection Agency (EPA) 2006]. Here, too, despite the institution of a standardized protocol for developmental neurotoxicity, few compounds have actually been tested relative to the total number of concern, even after mandated call-ins for data by the U.S. EPA (Makris 2006; U.S. EPA 2006).

A number of factors contribute to the dearth of information on developmental neurotoxicity relative to the demonstrated need for such knowledge. First, there is the essential dichotomy between the requirement to evaluate large numbers of compounds and the costly, cumbersome protocols prescribed for standard tests in animals (Colborn 2006; Makris 2006; Slotkin 2004b; U.S. EPA 2006). Second, testing compounds one at a time may produce results that are difficult to compare (Colborn 2006; U.S. EPA 2006), in large measure because the presumed mechanisms and targets are based on systemic or central nervous system effects in adults that may be unrelated to developmental neurotoxicity (Colborn 2006; Makris 2006; Slotkin 2004b, 2005). The organophosphate insecticides provide an archetype. Although it was originally thought that all organophosphates act similarly through irreversible inhibition of acetylcholinesterase, it is now evident that their adverse effects on brain development actually involve multiple mechanisms, many of which are unrelated to cholinesterase inhibition (Casida and Quistad 2004; Costa 2006; Pope 1999; Slotkin 2004a, 2004b, 2005). Accordingly, the relative potencies of organophosphates toward cholinesterase inhibition and/or systemic toxicity do not necessarily correlate with their propensity to elicit developmental neurotoxicity (Costa 2006; Pope 1999; Qiao et al. 2001; Slotkin et al. 2006a, 2006b).

One strategy proposed to resolve this problem is the use of screening techniques based on cell culture systems or lower organisms as the first stage of evaluation, thus enabling subsequent animal studies to focus on those compounds most likely to cause developmental neurotoxicity (Costa 1998; Slotkin 2004b). This approach was recently endorsed in a report from the Inspector General of the U.S. EPA (U.S. EPA 2006) as well as by outside groups (Colborn 2006; Costa 1998, 2006; Slotkin 2004b). In the present study we use PC12 cells, a standard *in vitro* model for neuronal development (Teng and Greene 1994) that has already been used to characterize essential features of the developmental neurotoxicity of organophosphates (Bagchi et al. 1995, 1996; Crumpton et al. 2000a, 2000b; Das and Barone 1999; Flaskos et al. 1994; Jameson et al. 2006b; Li and Casida 1998; Nagata et al. 1997; Qiao et al. 2001, 2005; Song et al. 1998; Tuler et al. 1989). As transformed cells, the PC12 line has an advantage over cultured primary neurons, which do not maintain cell division and thus cannot detect adverse effects on the cell cycle, a likely neurotoxic target; furthermore, primary neurons do not provide a uniform population either in terms of cell types or differentiation state, rendering their use for screening problematic. Upon exposure to nerve growth factor (NGF), PC12 cells gradually exit the mitotic cycle and begin to differentiate, developing axonal projections, electrical excitability, and the characteristics of two distinct phenotypes, cholinergic and catecholaminergic neurons (Fujita et al. 1989; Song et al. 1998; Teng and Greene 1994). Accordingly, the PC12 model enables the detection of toxicant actions that target cell replication as well as the events involved in differentiation and the phenotypic emergence of specific neuronal features. The PC12 model has been used to characterize the potential neurotoxicity of a wide variety of compounds in addition to the organophosphates, including nicotine, metals, and organometals (Abreu-Villaça et al. 2005; Benters et al. 1996; Crumpton et al. 2001; Matsuoka and Igisu 1996; Parran et al. 2003; Shafer 1998; Tian et al. 2000); largely, these have been done one compound or class at a time and with a focus on individual cellular targets or processes, rather than within a framework of comparative changes with a global impact on neurodevelopment. Critical for the use of PC12 cells in modeling the developmental neurotoxicity of compounds such as the organophosphates, the cholinergic and catecholaminergic phenotypes are among the prominent *in vivo* targets for these compounds (Aldridge et al. 2005; Barone et al. 2000; Dam et al. 1999; Jameson et al. 2006b; Pope 1999; Rice and Barone 2000; Slotkin 2004a; Slotkin et al. 2002; Vidair 2004). Nevertheless, PC12 cells share the limitations common to *in vitro* models, namely an inability to assess neuronal–glial interactions or architectural aspects of regional development, maternal–fetal or neonatal pharmacokinetics, and related issues of bioavailability, dose, and bioeffective concentrations (Costa 1998; Slotkin 2004b).

Although chlorpyrifos has been the most studied compound in the PC12 model (Bagchi et al. 1995, 1996; Crumpton et al. 2000a, 2000b; Das and Barone 1999; Jameson et al. 2006b; Qiao et al. 2001, 2005; Song et al. 1998), similarities and differences have been noted for other organophosphates such as diazinon and for nonorganophosphate cholinesterase inhibitors of the carbamate class, with physostigmine as the prototype (Jameson et al. 2006b; Qiao et al. 2001). In the present work, we compared chlorpyrifos, diazinon, and a third organophosphate (parathion) with physostigmine, an organochlorine (dieldrin), and a metal (Ni^2+^).These additional compounds were chosen for specific mechanistic and environmental reasons. First, all of them except physostigmine appear on the registry of Superfund Chemicals (U.S. National Library of Medicine 2006) and thus represent significant disposal problems. For diazinon, exposures of inner-city women during pregnancy are comparable to those seen with chlorpyrifos (Whyatt et al. 2002). The developmental neurotoxicity of diazinon has been studied sparingly, but shows a spectrum of effects similar to chlorpyrifos in both the PC12 model (Axelrad et al. 2002; Qiao et al. 2001) and in evaluations with lower organisms (Morale et al. 1998), albeit with a potency profile differing from the comparative effects of the two organophosphates on cholinesterase (Slotkin et al. 2006a). For parathion, despite U.S. restrictions, use and exposure remain common in agricultural communities around the world (Fenske et al. 2002). With chronic exposure to frankly toxic doses, parathion inhibits protein synthesis in the fetus (Gupta et al. 1984), whereas at lower exposures, it displays developmental toxicity that is not dependent on cholinesterase inhibition per se (Atterberry et al. 1997) but rather reflects differences in neural adaptations to exposure (Howard and Pope 2002; Karanth and Pope 2003; Liu et al. 1999). However, compared with chlorpyrifos and diazinon, parathion exhibits greater systemic toxicity relative to its propensity to produce developmental neurotoxicity (Slotkin et al. 2006a), again echoing the view that organophosphates are distinct in their profiles for adverse effects on the immature brain. Although the effects of parathion have not been evaluated in PC12 cells in a developmental context, other neural culture systems have successfully recapitulated the adverse effects on neurodevelopment and confirmed its dissociation from mechanisms involving cholinesterase inhibition (Monnet-Tschudi et al. 2000; Zurich et al. 2000). The carbamate physostigmine is effective as a cholinesterase inhibitor and shares some organophosphate-like effects on cell differentiation, but it is much less capable of eliciting immediate antimitotic actions (Jameson et al. 2006b; Qiao et al. 2001); it is considerably less effective than organophosphates as a developmental neurotoxicant in lower organisms (Buznikov et al. 2003).

In contrast to the organophosphates, organochlorines such as dieldrin have been less studied for developmental neurotoxicity, but the available evidence suggests a much more restricted range of mechanisms. Acute dieldrin intoxication produces fetal neural damage (Uzoukwu and Sleight 1972), and at lower concentrations, dieldrin interacts with γ-amino butyric acid (GABA_A_) channels in the fetal brain (Brannen et al. 1998); however, the long-term consequences of these lower-dose effects have not been evaluated. We suspect that the same strategies adopted for studies of organophosphates at the cellular level might prove useful in uncovering biomarkers and mechanisms for developmental neurotoxicity of dieldrin; for example, short-term, high-concentration dieldrin exposure of PC12 cells elicits oxidative stress and apoptosis differentially according to neurotransmitter phenotype (Kitazawa et al. 2001, 2003). Similarly, nickel compounds readily cross the placenta and accumulate in fetal tissues, including the brain, at concentrations that greatly exceed maternal levels (Jacobsen et al. 1978). Indeed, the nickel concentration just from normal dietary and environmental exposure in human fetuses is comparable to that of lead, up to 2 μg/g dry weight in soft tissues including the brain (Casey and Robinson 1978). Although the developmental neurotoxicity of nickel is almost completely unexplored, there is every reason to believe this metal may be as injurious as lead. In PC12 cells, Ni^2+^ interferes with the gating of calcium just as does lead or cadmium (Benters et al. 1996) in a manner that is dependent on the state of differentiation of the cells, so that the inclusion or exclusion of NGF makes a great difference in susceptibility (Nikodijevic and Guroff 1992). In developing sea urchins, Ni^2+^ produces phenotypic abnormalities that bear some resemblance to those caused by the organophosphates (Buznikov et al. 2003; Hardin et al. 1992).

Our overall strategy was to focus on aspects of neurodevelopment that lend themselves to rapid screening and that permit ready comparisons of targets involving cell replication, growth, and differentiation. We compared the effects of different concentrations and durations of exposure of PC12 cells to chlorpyrifos, diazinon, parathion, physostigmine, dieldrin, and Ni^2+^ in undifferentiated and differentiated states, evaluating indices of cell replication (radiolabeled thymidine incorporation into DNA), cell number, cell growth, viability (trypan blue exclusion), and phenotype. Each neural cell contains only a single nucleus (Winick and Noble 1965), so that the DNA content (micrograms of DNA per culture dish in the present study) reflects the total number of cells (Song et al. 1998). Indices of growth were provided by measurements of protein subfractions related to cell size and membrane surface area (Jameson et al. 2006a; Thai et al. 1996). The total protein/DNA ratio rises with cell enlargement, and the membrane/total protein ratio falls as a consequence of the decreased surface-to-volume ratio. On the other hand, with the onset of differentiation, the development of neuritic projections necessitates a rise in the relative contribution of membrane proteins, so that the increase in the membrane/total protein ratio gives an indication of augmented membrane “complexity.” The effects on cell number, size, and cell surface area were compared to those on viability (evaluated by trypan blue exclusion) and lipid peroxidation (evaluated by thiobarbituric acid-reactive species; TBARS). To characterize the catecholaminergic and cholinergic phenotypes, we assessed the ratio of activities of tyrosine hydroxylase (TH) to choline acetyltransferase (ChAT), the respective biosynthetic enzymes for dopamine and acetylcholine (Teng and Greene 1994; Jameson et al. 2006a, 2006b).

## Materials and Methods

### Cell cultures

Because of the clonal instability of the PC12 cell line (Fujita et al. 1989), the experiments were performed on cells that had undergone fewer than five passages, and all studies were repeated several times with different batches of cells. As described previously (Crumpton et al. 2000a; Qiao et al. 2003; Song et al. 1998), PC12 cells (1721-CRL; American Type Culture Collection, Manassas, VA) were seeded onto 100-mm poly-d-lysine-coated plates in RPMI-1640 medium (Invitrogen, Carlsbad, CA) supplemented with 10% inactivated horse serum (Sigma Chemical Co., St. Louis, MO), 5% fetal bovine serum (Sigma Chemical Co.), and 50 μg/mL penicillin streptomycin (Invitrogen). Cells were incubated with 7.5% CO_2_ at 37°C, and the medium was changed every 2 days. For studies in the undifferentiated state, cells were seeded at varying densities so that, regardless of the total time of incubation, the cells would reach a final confluence of 60–70%. Twenty-four hours after seeding, the medium was changed to include the various test substances: chlorpyrifos (Chem Service, West Chester, PA), diazinon (Chem Service), parathion (Chem Service), physostigmine (Sigma Chemical Co.), dieldrin (Chem Service), or NiCl_2_ (Sigma Chemical Co.). Because of their poor water solubility, the pesticides were dissolved in dimethyl sulfoxide (Sigma Chemical Co.), achieving a final concentration of 0.1% in the culture medium; accordingly, all cultures included this vehicle, which had no effect on the PC12 cells (Qiao et al. 2001, 2003; Song et al. 1998).

For studies in differentiating cells, 3 × 106 cells were seeded; 24 hr later, the medium was changed to include 50 ng/mL 2.5 S murine NGF (Invitrogen), and each culture was examined under a microscope to verify the subsequent outgrowth of neurites. The test agents were added concurrently with the start of NGF treatment.

### DNA synthesis

To initiate the measurement of DNA synthesis, the medium was changed to include 1 μCi/mL of [^3^H]thymidine (specific activity, 2 Ci/mmol; GE Healthcare, Piscataway, NJ) along with the continued inclusion of the test substances. After 1 hr, the medium was aspirated and cells were harvested in ice-cold water. Duplicate aliquots of each sample were treated with 10% trichloroacetic acid and sedimented at 1,000 × *g* for 15 min to precipitate macromolecules. The resulting pellet was washed once with additional trichloroacetic acid and then with 75% ethanol. The final pellet was hydrolyzed with 1 M KOH overnight at 37°C and neutralized with 6 M HCl, and the DNA was precipitated with ice-cold 5% trichloro-acetic acid and resedimented. The supernatant solution, comprising solubilized RNA and protein, was discarded. The DNA-containing pellet was hydrolyzed in 5% trichloroacetic acid for 15 min at 90°C and resedimented, and an aliquot of the supernatant solution was counted for radiolabel. Another aliquot was assayed for DNA spectrophotometrically by absorbance at 260 nm. Previous work has demonstrated quantitative recovery of DNA by these techniques (Bell et al. 1986; Slotkin et al. 1984). Incorporation values were corrected to the amount of DNA present in each culture to provide an index of macromolecule synthesis per cell (Winick and Noble 1965).

### Cell number and size

For determinations of DNA content, total protein/DNA ratio and membrane/total protein ratio, the medium was aspirated and the culture was rinsed with a buffer consisting of 154 mM NaCl and 10 mM sodium phosphate (pH 7.4). Cells were harvested in ice-cold buffer and homogenized (Polytron; Brinkmann Instruments, Westbury, NY), and aliquots were withdrawn for measurements of DNA and total protein using dye-binding methods (Trauth et al. 2000). To prepare the cell membrane fraction, the homogenates were sedimented at 40,000 × *g* for 10 min and the pellet was washed and resedimented. Aliquots of the final resuspension were then assayed for membrane protein (Smith et al. 1985).

### Enzyme activities

Cells were harvested as described above and were disrupted by homogenization in a ground-glass homogenizer fitted with a ground-glass pestle, using a buffer consisting of 154 mM NaCl and 10 mM sodium-potassium phosphate (pH 7.4). Aliquots were withdrawn for measurement of DNA and protein (Smith et al. 1985).

ChAT assays (Lau et al. 1988) were conducted in 60 μL of a buffer consisting of 60 mM sodium phosphate (pH 7.9), 200 mM NaCl, 20 mM choline chloride, 17 mM MgCl_2_, 1 mM EDTA, 0.2% Triton X-100, 0.12 mM physostigmine, and 0.6 mg/mL bovine serum albumin (Sigma Chemical Co.), containing a final concentration of 50 μM [^14^C]acetyl-coenzyme A (specific activity 60 mCi/mmol, diluted with unlabeled compound to 6.7 mCi/mmol; PerkinElmer Life Sciences, Boston, MA). The amount of protein used in each assay was adjusted to maintain activity within the linear range. Blanks contained homogenization buffer instead of the tissue homogenate. Samples were pre-incubated for 15 min on ice and transferred to a 37°C water bath for 30 min; the reaction was terminated by placing the samples on ice. Labeled acetylcholine was then extracted and counted in a liquid scintillation counter and the activity was calculated as nanomoles synthesized per hour per microgram DNA.

TH activity was measured using [^14^C]tyrosine as a substrate and trapping the evolved ^14^CO_2_ after coupled decarboxylation with dopa decarboxylase (Lau et al. 1988; Waymire et al. 1971). Homogenates were sedimented at 26,000 × *g* for 10 min to remove storage vesicles containing catecholamines, which interfere with TH activity, and assays were conducted with 100 μL aliquots of the supernatant solution in a total volume of 550 μL. Each assay (pH 6.1) contained final concentrations of 910 μM FeSO_4_, 55 μM unlabeled L-tyrosine (Sigma Chemical Co.), 9.1 μM pyridoxal phosphate (Sigma Chemical Co.), 36 μM β-mercaptoethanol, and 180 μM 2-amino-6,7-dimethyl-4-hydroxy-5,6,7,8-tetrahydropteridine HCl (Sigma Chemical Co.), all in a buffer of 180 mM sodium acetate and 1.8 mM sodium phosphate (pH 6.1). Each assay contained 0.5 μCi of generally labeled [^14^C]tyrosine (specific activity, 438 mCi/mmol; Sigma Chemical Co.) as substrate, and blanks contained buffer in place of the homogenate. Activity was calculated on the same basis as for ChAT.

### Thiobarbituric acid-reactive species

TBARS determinations were carried out by a modification (Qiao et al. 2005) of published procedures (Guan et al. 2003). Cells were harvested with 150 mM NaCl and 10 mM sodium phosphate (pH 7.4) and sonicated for 20 sec; aliquots were withdrawn for measurements of DNA and protein (Smith et al. 1985; Trauth et al. 2000). Another aliquot was added to 0.5 volumes of 15% trichloroacetic acid, followed by 1.5 volumes of thiobarbituric acid reagent: 0.67 g thiobarbituric acid (Sigma Chemical Co.) dissolved in 80 mL of 1 M NaOH, titrated to pH 3.5 with 20 mL glacial acetic acid. Samples were incubated for 60 min at 90–100°C, cooled to room temperature, and sedimented at 3,000 × *g* for 10 min. The supernatant solution was resedimented and absorption was determined at 532 nm, using malonaldehyde bis(dimethylacetal) (Sigma Chemical Co.) as a standard. To give the TBARS concentration per cell, values were calculated relative to the amount of DNA.

### Viability

To assess cell viability, the cell culture medium was changed to include trypan blue (1 volume per 2.5 volumes of medium; Sigma Chemical Co.) and cells were examined for staining under 400× magnification, counting an average of 100 cells per field in four different fields per culture.

### Data analysis

Data are presented as means and standard errors. For each type of study, treatment differences were first evaluated with a global analysis of variance (ANOVA; data log-transformed whenever variance was heterogeneous) incorporating all variables: cell batch number, treatment, and time. Based on the main treatment effects and/or interactions of treatment × time, differences for individual treatments were evaluated post hoc by Fisher’s protected least significant difference. For all tests, significance was assumed at *p* < 0.05. In the initial test, the results did not vary among the different batches of cells, so results across the different batches were combined for presentation and the indicated number of samples in each experiment reflects the total number of cultures.

## Results

**Figure 1:**
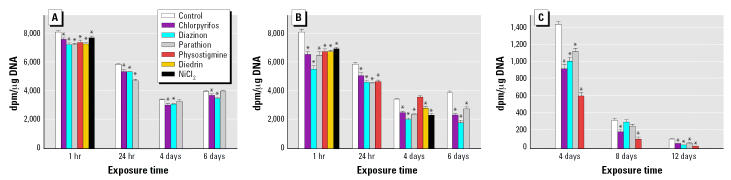
DNA synthesis shown as disintegrations per minute (dpm; mean ± SE) of [^3^H]thymidine in (*A*) undifferentiated cells exposed to 5 μM of agent, (*B*) undifferentiated cells exposed to 30 μM, and (*C*) differentiating cells exposed to 30 μM and cotreated with NGF (note that scale in *C* differs from that in *A* and *B*). ANOVA across all treatments and time points (number of determinations for each condition): (*A*), treatment, *p* < 0.0001; treatment × time, *p* < 0.0001 (*n* = 6–18); (*B*), treatment, *p* < 0.0001; treatment × time, *p* < 0.0001 (*n* = 12–30); (*C*), treatment, *p* < 0.0001; treatment × time, *p* < 0.0001 (*n* = 12–30). *Significantly different from the corresponding control value (*p* < 0.05).

In undifferentiated PC12 cells, even a 1-hr exposure to a low concentration (5 μM) of each of the agents elicited a small but statistically significant reduction in the rate of cell replication as monitored by the incorporation of [^3^H]thymidine into DNA (Figure 1A). Among the organophosphates, diazinon and parathion were slightly more effective than chlorpyrifos (e.g., at 1 hr of exposure, *p* < 0.03 for diazinon or parathion vs. chlorpyrifos); for the other agents, physostigmine and dieldrin were similar to diazinon, whereas Ni^2+^ was only about as effective as chlorpyrifos (*p* < 0.03 for Ni^2+^ vs. diazinon). We then evaluated whether the response was maintained with continued exposure to the organophosphates. After 24 hr exposure, all the compounds remained effective; nevertheless, the response to parathion disappeared after 4–6 days of continuous exposure, whereas the effects of chlorpyrifos and diazinon remained detectable. Raising the concentration to 30 μM produced correspondingly more robust inhibition of DNA synthesis in undifferentiated cells (Figure 1B). Again, diazinon remained more effective than chlorpyrifos with 1 hr of exposure (*p* < 0.0001), and this relationship remained over a span of 6 days. At this higher concentration, the effect of parathion was maintained throughout the exposure period. However, physostigmine showed adaptation by 4 days of exposure, whereas the adverse effects of the organophosphates, dieldrin, and Ni^2+^ remained fully in evidence.

In cells undergoing NGF-induced differentiation, the rate of DNA synthesis was far lower than in undifferentiated cells and declined precipitously over the span of 12 days (Figure 1C, note change in ordinate scale). Nevertheless, inhibitory effects of the organophosphates and physostigmine were fully in evidence after 4 days of coexposure with NGF. Unlike the situation in undifferentiated cells, the response to physostigmine did not show attenuation with continued exposure and, in fact, the carbamate had a greater effect than did the organophosphates (*p* < 0.02 vs. chlorpyrifos; *p* < 0.005 vs. diazinon; *p* < 0.0008 vs. parathion).

**Figure 2:**
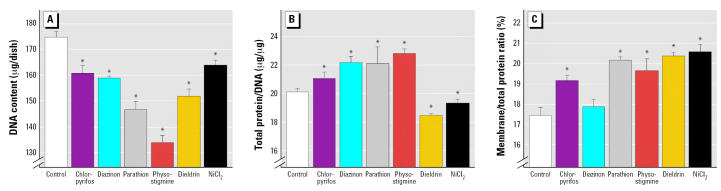
Indices of cell number and size (mean ± SE) in undifferentiated cells exposed to 30 μM of agent for 6 days. (*A*) DNA content. (*B*) Total protein/DNA ratio. (*C*) Membrane/total protein ratio. ANOVA across all treatments (number of determinations for each condition): (*A*) *p* < 0.0001 (*n* = 5–10); (*B*) *p* < 0.0001 (*n* = 5–10); (*C*) *p* < 0.0004 (*n* = 5–10). *Significantly different from the corresponding control value (*p* < 0.05).

In keeping with their ability to inhibit DNA synthesis, all the test agents produced significant shortfalls in cell number when undifferentiated cells were exposed for a period of 6 days (Figure 2A). Chlorpyrifos and diazinon each elicited a reduction of about 10%, whereas parathion was somewhat more effective (*p* < 0.01 vs. either chlorpyrifos or diazinon) and physostigmine substantially more so (*p* < 0.0001 vs. chlorpyrifos or diazinon; *p* < 0.004 vs. parathion). Dieldrin and Ni^2 +^ had effects similar to those of the organophosphates. In contrast, cell growth was not suppressed by the organophosphates or physostigmine and, in fact, there were significant elevations in the total protein/DNA ratio for these agents (Figure 2B). For this index, there were clear differences between these compounds and dieldrin or Ni^2+^, both of which evoked a significant reduction in the total protein/DNA ratio. In keeping with a reduction in cell size for these two agents, the membrane/total protein ratio rose, reflecting the higher surface/volume ratio associated with smaller cells (Figure 2C). Paradoxically, chlorpyrifos, parathion, and physostigmine increased the membrane/total protein ratio, despite the fact that they also increased the total protein/DNA ratio, implying that these agents actually augmented the membrane complexity of the cells as an accompaniment to the increase in cell size. Diazinon did not share these properties.

**Figure 3:**
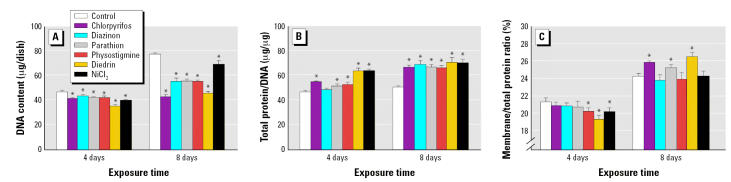
Indices of cell number and size (mean ± SE) in differentiating cells exposed to 30 μM of agent and cotreated with NGF. (*A*) DNA content. (*B*) Total protein/DNA ratio. (*C*) Membrane/total protein ratio (note the interrupted ordinate scale). ANOVA across all treatments and both time points (number of determinations for each condition): (*A*) treatment, *p* < 0.0001; treatment × time, *p* < 0.0001 (*n* = 10–22); (*B*) treatment, *p* < 0.0001; treatment × time, *p* < 0.0001 (*n* = 10–22); (*C*) treatment, *p* < 0.004; treatment × time, *p* < 0.0001 (*n* = 10–22). *Significantly different from the corresponding control value (*p* < 0.05).

We next conducted parallel studies for bio-markers of cell number and size in differentiating cells at 4 and 8 days of exposure. All of the agents elicited significant decrements in cell number across both time points (Figure 3A). With the exception of Ni^2+^, the adverse effects worsened substantially between 4 and 8 days and were much greater in magnitude than the deficits seen in undifferentiated cells (note different scales for Figure 2A and 3A). Again, we did not see evidence for additional impairment of cell growth: all agents increased the total protein/DNA ratio significantly, with a correspondingly greater effect at 8 days, when cell number was reduced the most (Figure 3B). On the other hand, there were biphasic effects on the membrane/total protein ratio in differentiating cells (Figure 3C). At 4 days of exposure, the organophosphates had little or no effect on this marker, whereas physostigmine, dieldrin, and Ni^2+^ all elicited a significant reduction. After 8 days of continuous exposure, the differentiating cells showed increases in the membrane/total protein ratio for chlorpyrifos, parathion, and dieldrin, whereas the other three agents had no significant effect.

**Figure 4:**
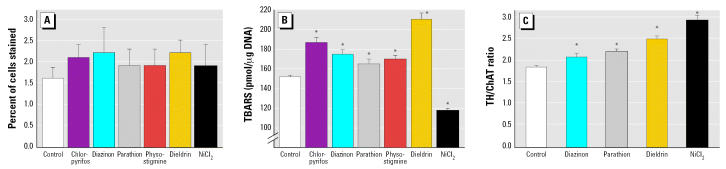
Indices of viability, oxidative damage, and transmitter phenotype (mean ± SE) in differentiating cells exposed to 30 μM of agent and cotreated with NGF for 6 days. (*A*) Trypan blue staining. (*B*) TBARS (note the interrupted ordinate scale). (*C*) TH/ChAT activity ratio. ANOVA across all treatments (number of determinations for each condition) (*A*) not significant (*n* = 10–22); (*B*) *p* < 0.0001 (*n* = 5–10); (*C*) *p* < 0.0001 (*n* = 10–25). *Significantly different from the corresponding control value (*p* < 0.05).

In differentiating cells after 6 days of co-exposure to NGF and each agent, there were no significant effects on trypan blue exclusion (Figure 4A) and the proportion of nonviable cells was uniformly low in all preparations. In contrast, we obtained evidence for significant membrane lipid peroxidation, as evidenced by the TBARS assay (Figure 4B). All of the organophosphates, as well as physostigmine and dieldrin, evoked significant elevations in TBARS, whereas Ni^2+^ uniquely caused a reduction. Nevertheless, all the agents produced a shift away from the cholinergic phenotype and toward the catecholaminergic phenotype, as evidenced by a significant increase in the TH/ChAT ratio (Figure 4C); similar results for chlorpyrifos and physostigmine have been published previously (Jameson et al. 2006b).

## Discussion

One of the major concerns about developmental neurotoxicity is the fact that the targeting of the immature brain may occur at toxicant exposures that are nonsymptomatic or that do not elicit general signs of systemic or cytotoxicity (Boyes 2001; U.S. EPA 2006). In the present study, we focused on exposures of PC12 cells to organophosphates, a carbamate, an organochlorine, and a metal that did not by themselves elicit general cytotoxic damage as monitored by trypan blue exclusion. Furthermore, even the 30 μM concentration used for most of our studies lies within the 100–1,000× safety factor required for establishing neurotoxic end points (Boyes 2001; U.S. EPA 2006); indeed, for the organophosphates, fetal exposures in agricultural communities are likely to be nearly as high (Ostrea et al. 2002), and similarly, even routine dietary intake produces brain Ni^2+^ concentrations approximating 10 μM (Casey and Robinson 1978). There are three essential findings in our study. First, a single agent may target multiple events in neural cell replication and differentiation, thus spanning a wide range of developmental stages. Second, otherwise unrelated chemicals that likely possess different originating mechanisms of action can nevertheless converge on a common set of final events in cell development, producing similar outcomes. Finally, and perhaps most importantly, our evaluations show the potential utility of an approach using neuronotypic cells in culture to screen suspected developmental neurotoxicants, enabling characterization of vulnerable stages, likely outcomes, and rank comparisons of related and unrelated chemicals.

In our earlier work with organophosphates, we demonstrated the ability of chlorpyrifos to cause immediate inhibition of cell replication in undifferentiated PC12 cells, exemplified by a reduction in [^3^H]thymidine incorporation into DNA within the first hour of exposure (Song et al. 1998), an effect that mirrors similar actions on the developing brain *in vivo* (Whitney et al. 1995) and that ultimately leads to deficits in neural cell numbers (Slotkin 2004a, 2004b, 2005), just as seen here in the PC12 model. The effect was shared by another organophosphate (diazinon), whereas a carbamate (physostigmine) was much less effective (Qiao et al. 2001). Importantly, chlorpyrifos oxon had a lesser action than chlorpyrifos, despite the fact that it is 1,000-fold more potent toward inhibition of cholinesterase (Das and Barone 1999). Furthermore, cholinergic antagonists failed to block the effect (Song et al. 1998), demonstrating that the adverse actions of organophosphates on cell replication were separate from anticholinesterase activity. Here, we expanded our findings to include another organophosphate (parathion), an organochlorine (dieldrin), and a metal (Ni^2+^); compared effects at a threshold concentration of 5 μM, as well as at 30 μM; determined the persistence over a course of nearly 1 week of exposure; and evaluated the downstream consequences for cell acquisition and growth. Although all the organophosphates evoked significant reductions in DNA synthesis, diazinon was the most effective over a prolonged time course: after 6 days of continuous exposure, diazinon maintained a significantly greater inhibitory effect than did either chlorpyrifos or parathion. Interestingly, this differs from the rank order of effects at 1 hr in PC12 cells incubated without the inclusion of serum proteins (Qiao et al. 2001), where chlorpyrifos is more effective than diazinon. It is important to note that the organophosphates show strong binding to serum proteins both *in vivo* and *in vitro* (Braeckman et al. 1983; Qiao et al. 2001), which reduces their bioeffective concentrations. The effect is highest for chlorpyrifos, less important for parathion, even lower for diazinon, and lowest for physostigmine (Braeckman et al. 1983; Sultatos et al. 1984; Whelpton and Hurst 1990; Wu et al. 1996). For the present study, serum proteins could not be deleted from the medium because they are required to maintain cell growth and viability, and consequently, the rank order of effects changes so that diazinon and physostigmine, with their lower binding, exert greater net effects than would otherwise be expected. These are not unimportant details: the actions of the organophosphates and carbamates *in vivo* are clearly modified by their binding to serum proteins in the circulation. Also, the concentration of these proteins is lower in the fetus than in the adult (Thom et al. 1967; Yaffe and Stern 1976), so that at comparable concentrations of each neurotoxicant, the fetus will bear a disproportionate burden of adverse effects. Additional factors must be operating to distinguish among the effects of the different organophosphates. For example, we found that the ability of parathion to reduce DNA synthesis at a low concentration (5 μM) showed eventual adaptation, so by 4–6 days of continuous exposure, its inhibitory actions were no longer evident. In contrast, chlorpyrifos and diazinon showed persistence of the effect. Again, these findings illustrate that the PC12 model can incorporate factors such as protein binding that are critical issues for the developmental neurotoxicity of disparate compounds *in vivo*. Based on these findings, it might be anticipated that the various organophosphates will differ from each other and from the carbamates in their adverse effects on brain development, a prediction that should be pursued in future studies.

Given that organophosphates reduce DNA synthesis in undifferentiated cells in a manner different from their ability to inhibit cholinesterase (Qiao et al. 2001; Song et al. 1998), it is of considerable import that we obtained a similar result with either dieldrin or Ni^2+^, compounds otherwise unrelated to each other, the organophosphates, or physostigmine. This, too, illustrates how disparate neurotoxicants can nevertheless converge on a common set of functional end points and thus may share many of the same attributes for adverse effects on the developing brain *in vivo*. In the present study, the consequences were examined through a series of markers related to cell acquisition and growth. In the undifferentiated cells, 6 days of continuous exposure to each of the agents produced a corresponding decrement in the total number of cells as monitored by DNA content. However, the rank order did not correspond one-to-one with the relative effects on DNA synthesis. For cell acquisition, physostigmine had the greatest adverse effect, followed by parathion, dieldrin, and then by chlorpyrifos ≈diazinon ≈Ni^2+.^ Accordingly, there must be other actions of these agents that influence the total number of cells other than their effect on DNA synthesis. These may include other, rate-limiting steps in the production of new cells downstream from DNA synthesis per se, as well as effects on cell turnover and/or apoptotic loss. We did not observe any decreases in viability as monitored with trypan blue, but a small, persistent adverse effect could eventually influence cell number without being detectable with this technique. Studies are currently under way to examine potentially subtle cytotoxic effects or apoptotic events using gene expression profiling, an approach that may be successful in elucidating these additional mechanisms. However, again, the main point is that a relatively facile set of markers in PC12 cells can detect adverse effects on neural cell acquisition across a wide array of disparate compounds in a short period of time.

In contrast to their effects on cell number, the organophosphates and physostigmine did not suppress cell growth in undifferentiated PC12 cells, as monitored by the protein/DNA ratio; in fact, each of these agents evoked a small but statistically significant increase. On the other hand, both dieldrin and Ni^2+^ did reduce the ratio, implying that these agents simultaneously suppress neural cell acquisition and cell growth. Again, this *in vitro* test system now points the way toward end points that may differentiate the developmental neurotoxicity of these two agents from those of the organophosphates or carbamates. Finally, the assessment of membrane complexity in undifferentiated cells (membrane/total protein ratio) indicated further differences among the agents, with significant increases for all except diazinon. For dieldrin and Ni^2+^, the increase in the ratio reflects in part the inhibition of cell growth, because the geometry of a smaller cell necessitates a higher surface-to-volume ratio. However, that cannot explain the increase for chlorpyrifos, parathion, and physostigmine, which did not suppress cell growth; for these agents, either intracellular organelles are being induced or, alternatively, they may provide a prodifferentiation signal that elicits expansion of the membrane surface as a prelude to neurite outgrowth. Again, this is in keeping with some of the known effects of chlorpyrifos, which can promote dendritic arborization at the expense of axon formation (Howard et al. 2005); our findings suggest that diazinon may be distinctly different from chlorpyrifos in that regard. Potentially, these possibilities can be resolved with detailed morphologic techniques, but those hardly lend themselves to rapid screening.

With the initiation of differentiation upon addition of NGF to the cultures, the spectrum of actions of the various agents underwent a distinct transition. For DNA synthesis, the rate of [^3^H]thymidine incorporation fell precipitously, as would be expected from the transition from cell replication to differentiation. During the transition, we again compared the organophosphates to physostigmine; although all the agents still produced significant inhibition of DNA synthesis, physostigmine now became the most effective of the agents and, unlike the situation in undifferentiated cells, did not show an adaptive loss of effect. This pattern is consistent with *in vivo* findings, which indicate that the effects of organophosphates on DNA synthesis undergo a switch from noncholinergic to cholinergic mechanisms as differentiation proceeds (Slotkin 1999, 2004a, 2004b, 2005; Whitney et al. 1995). Physostigmine, like the organophosphates, is a cholinesterase inhibitor; therefore, under these circumstances, the most relevant factor may simply be the much lower binding of physostigmine to serum proteins, which makes its bioeffective concentration higher than that of the organophosphates (Whelpton and Hurst 1990). Despite the fact that the overall rate of DNA synthesis was far lower in differentiating cells, prolonged exposure to any of the agents reduced the total number of cells as monitored by DNA content. Again, the effect on DNA synthesis could not explain the rank order of effects on cell number, for which the greatest effects were seen with chlorpyrifos and dieldrin, each of which reduced cell number by about 40%, followed by diazinon, parathion and physostigmine, with Ni^2+^ providing the smallest reduction. Notably, despite the fact that the DNA synthetic rate was lower in differentiating cells than in the undifferentiated state, the magnitude of the effects on cell number were greater during differentiation and, consequently, other factors contribute to the cell deficits. In earlier work with chlorpyrifos, we showed that the peak period of sensitivity occurs at the initiation of differentiation, where actions are exerted simultaneously through noncholinergic and cholinergic mechanisms (Jameson et al. 2006b; Qiao et al. 2001). Accompanying the reduction in cell numbers, there was an increase in cell size (protein/DNA ratio) for each agent, again suggesting that in differentiating cells—as in undifferentiated cells—there is no direct suppression of cell growth, nor was there evidence for loss of viability in the trypan blue test. In earlier work with chlorpyrifos, we found that inhibition of cell growth can occur, but only at higher concentrations than those used here (Song et al. 1998). However, for the membrane/total protein ratio, the nonorganophosphate agents caused an initial reduction in differentiating cells; because this was superimposed on an increase in protein/DNA, the results are consistent with larger cell bodies. With continued exposure, this effect disappeared, and for three agents (chlorpyrifos, parathion, dieldrin), there were increases in the membrane/total protein ratio. Obviously, then, superimposed on the deficits in cell number, there are changes in membrane complexity as differentiation proceeds, in a manner consistent with targeting of intracellular organelles and/or neuritic outgrowth. As discussed for some of the other findings, resolution of these issues requires morphologic determinations, which are thus not amenable to rapid screening; at the same time, the fact that these types of changes are detectable at the biochemical level lends support to the use of such indices. Notably, these measures strengthen the view that there are distinct differences among the various organophosphates, with diazinon showing unique properties; yet, at the same time, there is a resemblance of outcomes between some of the organophosphates and developmental neurotoxicants from different classes of compounds.

We conducted two additional sets of studies in differentiating cells that reinforce these conclusions. First, we examined TBARS as an index of lipid peroxidation with the idea that oxidative stress and resultant membrane damage could represent a common mechanism underlying the neurotoxicant actions of otherwise disparate compounds, especially in light of the prominence of oxidative stress in the noncholinesterase-related targets of organophosphates (Gupta 2004). Indeed, all of the agents except Ni^2+^ showed a significant increase in TBARS after 6 days in culture. Uniquely, Ni^2+^ reduced TBARS, perhaps because nickel can exist in higher oxidation states, thus acting as a reductant in the culture system. Accordingly, taken in isolation, lipid peroxidation cannot explain commonalities between neurotoxicant end points of Ni^2+^ and the other agents. Nevertheless, it should be noted that earlier effects may go unnoticed and that we did not assess oxidative damage to cellular components other than membrane lipids (e.g., DNA). Again, the principle is that we are examining those aspects of neurodevelopment that lend themselves to rapid screening rather than investigating every potential mechanism of action.

The second set of studies concerned effects on the neurotransmitter phenotypic fate of the cells. In earlier work, we found that both chlorpyrifos and physostigmine suppress the expression of the cholinergic phenotype in favor of the catecholaminergic phenotype, thus raising the TH/ChAT ratio (Jameson et al. 2006b). The same change in catecholaminergic/cholinergic balance occurs with chlorpyrifos treatment *in vivo* (Dam et al. 1999; Meyer et al. 2004; Slotkin 2004a; Slotkin et al. 2005). Here, we found the same effects for all of the agents, with Ni^2+^ producing the largest shift in phenotype. Given the disparate nature of the various compounds tested here, it is highly unlikely that they all share the same specific targeting of gene promoters for the two phenotypes and rather, it appears that the cholinergic phenotype is simply far more vulnerable to neurotoxicant actions than is the catecholaminergic phenotype. In turn, this may explain why a wide variety of developmental neurotoxicants all seem to produce a similar pattern of cognitive defects that center around cholinergic synaptic function in key areas such as the hippocampus (Eriksson 1997; Hohmann and Berger-Sweeney 1998; Morley and Happe 2000; Slotkin 2004a; Wessler et al. 1999; Yanai et al. 2002, 2004). The TH/ChAT index also supports the ability of the PC12 test system to readily distinguish toxicant effects that alter transmitter choice during neural cell differentiation, an effect that could readily contribute to the “miswiring” of key brain areas and resultant neurobehavioral deficits.

In conclusion, our results show the utility of the PC12 cell model as a potential screen for developmental neurotoxicants. This cell line allows for distinctions to be made between effects exerted on cell replication as compared to the more complex panoply of events occurring during differentiation, and can be applied to completely different classes of compounds. For the agents examined here, we were able to distinguish rank-order effects among different organophosphates and similarities and differences from a carbamate, which, although not an organophosphate, shares the ability to inhibit cholinesterase. Perhaps equally valuable, we showed how many features of developmental neurotoxicity are shared by an organochlorine (dieldrin) and a metal (Ni^2+^), thus leading to a number of predictions about the effects of these agents on brain development that can be tested *in vivo*. Nevertheless, it is important to distinguish between the use of cell lines that may be useful for screening of neurotoxicants and the potential for high-throughput techniques that would actually be required to have an impact for evaluation of the developmental neurotoxicity of the thousands of new chemical entities released each year. With regard to the end points used here, the radiometric techniques would need to be replaced by optical methods that lend themselves to detection with assay robots; certainly, there are far more potential end points of interest than those evaluated in the present study, such as apoptosis, development of receptors and ion channels, and dendritic arborization. Nor would it suffice to use only the PC12 model, as other cell systems may be required to evaluate the panoply of potential neurotoxic effects. Instead, a stepwise screening procedure is likely to be the best approach (Colborn 2006; Costa 1998, 2006; Slotkin 2004b): cell culture results can be followed by rapid screening in lower organisms such as zebrafish (Linney et al. 2004) and sea urchin (Buznikov et al. 2003), thus reducing the number of chemicals that ultimately need to be tested in mammals and narrowing the focus to enable better detection of the likely targets and end points in the mammalian brain. The results presented here, even though they involve only a handful of test compounds and end points in a single cell line, demonstrate the feasibility of one step in the development of a rapid *in vitro* screening procedure that may ultimately enable this type of sequential approach to tackle the problem of evaluating the thousands of potentially neurotoxic chemicals in the environment.

## References

[b1-ehp0115-000093] Abreu-Villaça Y, Seidler FJ, Qiao D, Slotkin TA (2005). Modeling the developmental neurotoxicity of nicotine in vitro: cell acquisition, growth and viability in PC12 cells. Dev Brain Res.

[b2-ehp0115-000093] Aldridge JE, Meyer A, Seidler FJ, Slotkin TA (2005). Alterations in central nervous system serotonergic and dopaminergic synaptic activity in adulthood after prenatal or neonatal chlorpyrifos exposure. Environ Health Perspect.

[b3-ehp0115-000093] Atterberry TT, Burnett WT, Chambers JE (1997). Age-related differences in parathion and chlorpyrifos toxicity in male rats: target and nontarget esterase sensitivity and cytochrome P450-mediated metabolism. Toxicol Appl Pharmacol.

[b4-ehp0115-000093] Axelrad JC, Howard CV, McLean WG (2002). Interactions between pesticides and components of pesticide formulations in an in vitro neurotoxicity test. Toxicology.

[b5-ehp0115-000093] Bagchi D, Bagchi M, Hassoun EA, Stohs SJ (1995). In vitro and in vivo generation of reactive oxygen species, DNA damage and lactate dehydrogenase leakage by selected pesticides. Toxicology.

[b6-ehp0115-000093] Bagchi D, Bhattacharya G, Stohs SJ (1996). In vitro and in vivo induction of heat shock (stress) protein (Hsp) gene expression by selected pesticides. Toxicology.

[b7-ehp0115-000093] Barone S, Das KP, Lassiter TL, White LD (2000). Vulnerable processes of nervous system development: a review of markers and methods. Neurotoxicology.

[b8-ehp0115-000093] Bell JM, Whitmore WL, Slotkin TA (1986). Effects of α-difluoro-methylornithine, a specific irreversible inhibitor of ornithine decarboxylase, on nucleic acids and proteins in developing rat brain: critical perinatal periods for regional selectivity. Neuroscience.

[b9-ehp0115-000093] Benters J, Schafer T, Beyersmann D, Hechtenberg S (1996). Agonist-stimulated calcium transients in PC12 cells are affected differentially by cadmium and nickel. Cell Calcium.

[b10-ehp0115-000093] Boyes WK 2001. Neurotoxicology and behavior. In: Patty’s Toxicology (Bingham E, Cohrseen B, Powell CH, eds). 5th ed. New York:John Wiley & Sons, 55–121.

[b11-ehp0115-000093] Braeckman RA, Audenaert F, Willems JL, Belpaire FM, Bogaert MG (1983). Toxicokinetics of methyl parathion and parathion in the dog after intravenous and oral administration. Arch Toxicol.

[b12-ehp0115-000093] Brannen KC, Devaud LL, Liu J, Lauder JM (1998). Prenatal exposure to neurotoxicants dieldrin or lindane alters *tert*-butyl-bicyclophosphorothionate binding to GABA_A_ receptors in fetal rat brainstem. Dev Neurosci.

[b13-ehp0115-000093] Buznikov GA Slotkin TA Lauder JM 2003. Sea urchin embryos and larvae as biosensors for neurotoxins. In: Current Protocols in Toxicology (Maines MD, Costa LG, Hodgson E, Reed DJ, eds). New York:John Wiley & Sons, 1.6.1–1.6.24. 10.1002/0471140856.tx0106s16 23045086

[b14-ehp0115-000093] Casey CE, Robinson MF (1978). Copper, manganese, zinc, nickel, cadmium and lead in human foetal tissues. Br J Nutrition.

[b15-ehp0115-000093] Casida JE, Quistad GB (2004). Organophosphate toxicology: safety aspects of nonacetylcholinesterase secondary targets. Chem Res Toxicol.

[b16-ehp0115-000093] Claudio L, Kwa WC, Russell AL, Wallinga D (2000). Testing methods for developmental neurotoxicity of environmental chemicals. Toxicol Appl Pharmacol.

[b17-ehp0115-000093] Colborn T (2006). A case for revisiting the safety of pesticides: a closer look at neurodevelopment. Environ Health Perspect.

[b18-ehp0115-000093] Costa LG (1998). Neurotoxicity testing: a discussion of *in vitro* alternatives. Environ Health Perspect.

[b19-ehp0115-000093] Costa LG (2006). Current issues in organophosphate toxicology. Clin Chim Acta.

[b20-ehp0115-000093] Crumpton T, Atkins DS, Zawia NH, Barone S (2001). Lead exposure in pheochromocytoma (PC12) cells alters neural differentiation and Sp1 DNA-binding. Neurotoxicology.

[b21-ehp0115-000093] Crumpton TL, Seidler FJ, Slotkin TA (2000a). Developmental neurotoxicity of chlorpyrifos in vivo and in vitro: effects on nuclear transcription factor involved in cell replication and differentiation. Brain Res.

[b22-ehp0115-000093] Crumpton TL, Seidler FJ, Slotkin TA (2000b). Is oxidative stress involved in the developmental neurotoxicity of chlorpyrifos?. Dev Brain Res.

[b23-ehp0115-000093] Dam K, Garcia SJ, Seidler FJ, Slotkin TA (1999). Neonatal chlorpyrifos exposure alters synaptic development and neuronal activity in cholinergic and catecholaminergic pathways. Dev Brain Res.

[b24-ehp0115-000093] Das KP, Barone S (1999). Neuronal differentiation in PC12 cells is inhibited by chlorpyrifos and its metabolites: is acetyl-cholinesterase inhibition the site of action?. Toxicol Appl Pharmacol.

[b25-ehp0115-000093] Eriksson P (1997). Developmental neurotoxicity of environmental agents in the neonate. Neurotoxicology.

[b26-ehp0115-000093] Fenske RA, Lu CS, Barr D, Needham L (2002). Children’s exposure to chlorpyrifos and parathion in an agricultural community in central Washington State. Environ Health Perspect.

[b27-ehp0115-000093] Flaskos J, McLean WG, Hargreaves AJ (1994). The toxicity of organophosphate compounds towards cultured PC12 cells. Toxicol Lett.

[b28-ehp0115-000093] Fujita K, Lazarovici P, Guroff G (1989). Regulation of the differentiation of PC12 pheochromocytoma cells. Environ Health Perspect.

[b29-ehp0115-000093] Guan ZZ, Yu WF, Nordberg A (2003). Dual effects of nicotine on oxidative stress and neuroprotection in PC12 cells. Neurochem Int.

[b30-ehp0115-000093] Gupta RC (2004). Brain regional heterogeneity and toxicological mechanisms of organophosphates and carbamates. Toxicol Mech Meth.

[b31-ehp0115-000093] Gupta RC, Thornburg JE, Stedman DB, Welsch F (1984). Effect of subchronic administration of methyl parathion on *in vivo* protein synthesis in pregnant rats and their conceptuses. Toxicol Appl Pharmacol.

[b32-ehp0115-000093] Hardin J, Coffman JA, Black SD, McClay DR (1992). Commitment along the dorsoventral axis of the sea urchin embryo is altered in response to NiCl_2_. Development.

[b33-ehp0115-000093] Hohmann CF, Berger-Sweeney J (1998). Cholinergic regulation of cortical development and plasticity: new twists to an old story. Perspect Dev Neurobiol.

[b34-ehp0115-000093] Howard AS, Bucelli R, Jett DA, Bruun D, Yang DR (2005). Chlorpyrifos exerts opposing effects on axonal and dendritic growth in primary neuronal cultures. Toxicol Appl Pharmacol.

[b35-ehp0115-000093] Howard MD, Pope CN (2002). *In vitro* effects of chlorpyrifos, parathion, methyl parathion and their oxons on cardiac muscarinic receptor binding in neonatal and adult rats. Toxicology.

[b36-ehp0115-000093] Jacobsen N, Alfheim I, Jonsen J (1978). Nickel and strontium distribution in some mouse tissues. Passage through placenta and mammary glands. Res Comm Chem Pathol Pharmacol.

[b37-ehp0115-000093] Jameson RR, Seidler FJ, Qiao D, Slotkin TA (2006a). Adverse neurodevelopmental effects of dexamethasone modeled in PC12 cells: identifying the critical stages and concentration thresholds for the targeting of cell acquisition, differentiation and viability. Neuropsychopharmacology.

[b38-ehp0115-000093] Jameson RR, Seidler FJ, Qiao D, Slotkin TA (2006b). Chlorpyrifos affects phenotypic outcomes in a model of mammalian neurodevelopment: critical stages targeting differentiation in PC12 cells. Environ Health Perspect.

[b39-ehp0115-000093] Karanth S, Pope C (2003). Age-related effects of chlorpyrifos and parathion on acetylcholine synthesis in rat striatum. Neurotoxicol Teratol.

[b40-ehp0115-000093] Kitazawa M, Anantharam V, Kanthasamy AG (2001). Dieldrin-induced oxidative stress and neurochemical changes contribute to apoptopic cell death in dopaminergic cells. Free Radic Biol Med.

[b41-ehp0115-000093] Kitazawa M, Anantharam V, Kanthasamy AG (2003). Dieldrin induces apoptosis by promoting caspase-3-dependent proteolytic cleavage of protein kinase Cδin dopaminergic cells: relevance to oxidative stress and dopaminergic degeneration. Neuroscience.

[b42-ehp0115-000093] Landrigan PJ, Graham DG, Thomas RD (1994). Environmental neurotoxic illness: research for prevention. Environ Health Perspect.

[b43-ehp0115-000093] Lau C, Seidler FJ, Cameron AM, Navarro HA, Bell JM, Bartolome J (1988). Nutritional influences on adrenal chromaffin cell development: comparison with central neurons. Pediatr Res.

[b44-ehp0115-000093] Li WW, Casida JE (1998). Organophosphorus neuropathy target esterase inhibitors selectively block outgrowth of neurite-like and cell processes in cultured cells. Toxicol Lett.

[b45-ehp0115-000093] Linney E, Upchurch L, Donerly S (2004). Zebrafish as a neuro-toxicological model. Neurotoxicol Teratol.

[b46-ehp0115-000093] Liu J, Olivier K, Pope CN (1999). Comparative neurochemical effects of repeated methyl parathion or chlorpyrifos exposures in neonatal and adult rats. Toxicol Appl Pharmacol.

[b47-ehp0115-000093] Makris S 2006. Regulatory considerations in developmental neurotoxicity of organophosphorus and carbamate pesticides. In: Toxicity of Organophosphate and Carbamate Pesticides (Gupta RC, ed). San Diego:Elsevier Academic Press, 633–641.

[b48-ehp0115-000093] Matsuoka M, Igisu H (1996). Induction of c-*fos* expression by tributylin in PC12 cells: involvement of intracellular Ca^2+^. Environ Toxicol Pharmacol.

[b49-ehp0115-000093] Meyer A, Seidler FJ, Slotkin TA (2004). Developmental effects of chlorpyrifos extend beyond neurotoxicity: critical periods for immediate and delayed-onset effects on cardiac and hepatic cell signaling. Environ Health Perspect.

[b50-ehp0115-000093] Monnet-Tschudi F, Zurich MG, Schilter B, Costa LG, Honegger P (2000). Maturation-dependent effects of chlorpyrifos and parathion and their oxygen analogs on acetylcholinesterase and neuronal and glial markers in aggregating brain cell cultures. Toxicol Appl Pharmacol.

[b51-ehp0115-000093] Morale A, Coniglio L, Angelini C, Cimoli G, Bolla A, Alleteo D (1998). Biological effects of a neurotoxic pesticide at low concentrations on sea urchin early development: a teratogenic assay. Chemosphere.

[b52-ehp0115-000093] Morley BJ, Happe HK (2000). Cholinergic receptors: dual roles in transduction and plasticity. Hearing Res.

[b53-ehp0115-000093] Nagata K, Huang CS, Song JH, Narahashi T (1997). Direct actions of anticholinesterases on the neuronal nicotinic acetylcholine receptor channels. Brain Res.

[b54-ehp0115-000093] Nikodijevic B, Guroff G (1992). Nerve growth factor-stimulated calcium uptake into PC12 cells: uniqueness of the channel and evidence for phosphorylation. J Neurosci Res.

[b55-ehp0115-000093] Ostrea EM, Morales V, Ngoumgna E, Prescilla R, Tan E, Hernandez E (2002). Prevalence of fetal exposure to environmental toxins as determined by meconium analysis. Neurotoxicology.

[b56-ehp0115-000093] Parran DK, Barone S, Mundy WR (2003). Methylmercury decreases NGF-induced TrkA autophosphorylation and neurite outgrowth in PC12 cells. Dev Brain Res.

[b57-ehp0115-000093] Pope CN (1999). Organophosphorus pesticides: do they all have the same mechanism of toxicity?. J Toxicol Environ Health.

[b58-ehp0115-000093] Qiao D, Seidler FJ, Slotkin TA (2001). Developmental neurotoxicity of chlorpyrifos modeled *in vitro*: comparative effects of metabolites and other cholinesterase inhibitors on DNA synthesis in PC12 and C6 cells. Environ Health Perspect.

[b59-ehp0115-000093] Qiao D, Seidler FJ, Slotkin TA (2005). Oxidative mechanisms contributing to the developmental neurotoxicity of nicotine and chlorpyrifos. Toxicol Appl Pharmacol.

[b60-ehp0115-000093] Qiao D, Seidler FJ, Violin JD, Slotkin TA (2003). Nicotine is a developmental neurotoxicant and neuroprotectant: stage-selective inhibition of DNA synthesis coincident with shielding from effects of chlorpyrifos. Dev Brain Res.

[b61-ehp0115-000093] Rice D, Barone S (2000). Critical periods of vulnerability for the developing nervous system: evidence from humans and animal models. Environ Health Perspect.

[b62-ehp0115-000093] Shafer TJ (1998). Effects of Cd^2+^, Pb^2+^ and CH3Hg^+^ on high voltage-activated calcium currents in pheochromocytoma (PC12) cells: potency, reversibility, interactions with extracellular Ca^2+^ and mechanisms of block. Toxicol Lett.

[b63-ehp0115-000093] Slotkin TA (1999). Developmental cholinotoxicants: nicotine and chlorpyrifos. Environ Health Perspect.

[b64-ehp0115-000093] Slotkin TA (2004a). Cholinergic systems in brain development and disruption by neurotoxicants: nicotine, environmental tobacco smoke, organophosphates. Toxicol Appl Pharmacol.

[b65-ehp0115-000093] Slotkin TA (2004b). Guidelines for developmental neurotoxicity and their impact on organophosphate pesticides: a personal view from an academic perspective. Neurotoxicology.

[b66-ehp0115-000093] Slotkin TA 2005. Developmental neurotoxicity of organophosphates: a case study of chlorpyrifos. In: Toxicity of Organophosphate and Carbamate Pesticides (Gupta RC, ed). San Diego:Elsevier Academic Press, 293–314.

[b67-ehp0115-000093] Slotkin TA, Levin ED, Seidler FJ (2006a). Comparative developmental neurotoxicity of organophosphate insecticides: effects on brain development are separable from systemic toxicity. Environ Health Perspect.

[b68-ehp0115-000093] Slotkin TA, Persons D, Slepetis RJ, Taylor D, Bartolome J (1984). Control of nucleic acid and protein synthesis in developing brain, kidney, and heart of the neonatal rat: effects of α-difluoromethylornithine, a specific, irreversible inhibitor of ornithine decarboxylase. Teratology.

[b69-ehp0115-000093] Slotkin TA, Tate CA, Cousins MM, Seidler FJ (2002). Functional alterations in CNS catecholamine systems in adolescence and adulthood after neonatal chlorpyrifos exposure. Dev Brain Res.

[b70-ehp0115-000093] Slotkin TA, Tate CA, Cousins MM, Seidler FJ (2005). Imbalances emerge in cardiac autonomic cell signaling after neonatal exposure to terbutaline or chlorpyrifos, alone or in combination. Dev Brain Res.

[b71-ehp0115-000093] Slotkin TA, Tate CA, Ryde IT, Levin ED, Seidler FJ (2006b). Organophosphate insecticides target the serotonergic system in developing rat brain regions: disparate effects of diazinon and parathion at doses spanning the threshold for cholinesterase inhibition. Environ Health Perspect.

[b72-ehp0115-000093] Smith PK, Krohn RI, Hermanson GT, Mallia AK, Gartner FH, Provenzano MD (1985). Measurement of protein using bicinchoninic acid. Anal Biochem.

[b73-ehp0115-000093] Song X, Violin JD, Seidler FJ, Slotkin TA (1998). Modeling the developmental neurotoxicity of chlorpyrifos *in vitro*: macromolecule synthesis in PC12 cells. Toxicol Appl Pharmacol.

[b74-ehp0115-000093] Sultatos LG, Basker KM, Shao M, Murphy SD (1984). The interaction of the phosphorothioate insecticides chlorpyrifos and parathion and their oxygen analogues with bovine serum albumin. Mol Pharmacol.

[b75-ehp0115-000093] Szpir M (2006). New thinking on neurodevelopment. Environ Health Perspect.

[b76-ehp0115-000093] Teng KK Greene LA 1994. Cultured PC12 cells: a model for neuronal function and differentiation. In: Cell Biology: A Laboratory Handbook (Celis JE, ed). San Diego:Academic Press, 218–224.

[b77-ehp0115-000093] Thai L, Galluzzo JM, McCook EC, Seidler FJ, Slotkin TA (1996). Atypical regulation of hepatic adenylyl cyclase and adrenergic receptors during a critical developmental period: agonists evoke supersensitivity accompanied by failure of receptor downregulation. Pediatr Res.

[b78-ehp0115-000093] Thom H, McKay E, Gray DW (1967). Protein concentrations in the umbilical cord plasma of premature and mature infants. Clin Sci.

[b79-ehp0115-000093] Tian X, Sun X, Suszkiw JB (2000). Upregulation of tyrosine hydroxylase and downregulation of choline acetyltransferase in lead-exposed PC12 cells: the role of PKC activation. Toxicol Appl Pharmacol.

[b80-ehp0115-000093] Tilson HA (1995). The concern for developmental neurotoxicology: is it justified and what is being done about it?. Environ Health Perspect.

[b81-ehp0115-000093] Tilson HA (2000). Neurotoxicology risk assessment guidelines: developmental neurotoxicology. Neurotoxicology.

[b82-ehp0115-000093] Trauth JA, Seidler FJ, Slotkin TA (2000). An animal model of adolescent nicotine exposure: effects on gene expression and macromolecular constituents in rat brain regions. Brain Res.

[b83-ehp0115-000093] Tuler SM, Hazen AA, Bowen JM (1989). Release and metabolism of dopamine in a clonal line of pheochromocytoma (PC12) cells exposed to fenthion. Fundam Appl Toxicol.

[b84-ehp0115-000093] U.S. EPA (U.S. Environmental Protection Agency). 2006. Opportunities to Improve Data Quality and Children’s Health Through the Food Quality Protection Act. Report no http://www.epa.gov/oig/reports/2006/20060110-2006-P-00009.pdf.

[b85-ehp0115-000093] U.S. National Library of Medicine http://toxmap.nlm.nih.gov/toxmap/main/sfChemicals.jsp.

[b86-ehp0115-000093] Uzoukwu M, Sleight SD (1972). Dieldrin toxicosis: fetotoxicosis, tissue concentrations, and microscopic and ultrastructural changes in guinea pigs. Am J Vet Res.

[b87-ehp0115-000093] Vidair CA (2004). Age dependence of organophosphate and carbamate neurotoxicity in the postnatal rat: extrapolation to the human. Toxicol Appl Pharmacol.

[b88-ehp0115-000093] Waymire JC, Bjur R, Weiner N (1971). Assay of tyrosine hydroxylase by coupled decarboxylation of dopa formed from 1-^14^C-L-tyrosine. Anal Biochem.

[b89-ehp0115-000093] Wessler I, Kirkpatrick CJ, Racke K (1999). The cholinergic ‘pitfall’: acetylcholine, a universal cell molecule in biological systems, including humans. Clin Exp Pharmacol Physiol.

[b90-ehp0115-000093] Whelpton R, Hurst PR (1990). The binding of physostigmine to human serum albumin. J Pharm Pharmacol.

[b91-ehp0115-000093] Whitney KD, Seidler FJ, Slotkin TA (1995). Developmental neurotoxicity of chlorpyrifos: cellular mechanisms. Toxicol Appl Pharmacol.

[b92-ehp0115-000093] Whyatt RM, Camann DE, Kinney PL, Reyes A, Ramirez J, Dietrich J (2002). Residential pesticide use during pregnancy among a cohort of urban minority women. Environ Health Perspect.

[b93-ehp0115-000093] Winick M, Noble A (1965). Quantitative changes in DNA, RNA and protein during prenatal and postnatal growth in the rat. Dev Biol.

[b94-ehp0115-000093] Wu HX, Evreu-Gros C, Descotes J (1996). Diazinon toxicokinetics, tissue distribution and anticholinesterase activity in the rat. Biomed Environ Sci.

[b95-ehp0115-000093] Yaffe SJ Stern L 1976. Clinical implications of perinatal pharmacology. In: Perinatal Pharmacology and Therapeutics (Mirkin BL, ed). New York:Academic Press, 355–428.

[b96-ehp0115-000093] Yanai J, Beer A, Huleihel R, Izrael M, Katz S, Levi Y (2004). Convergent effects on cell signaling mechanisms mediate the actions of different neurobehavioral teratogens: alterations in cholinergic regulation of PKC in chick and avian models. Ann NY Acad Sci.

[b97-ehp0115-000093] Yanai J, Vatury O, Slotkin TA (2002). Cell signaling as a target and underlying mechanism for neurobehavioral teratogenesis. Ann NY Acad Sci.

[b98-ehp0115-000093] Zurich MG, Honegger P, Schilter B, Costa LG, Monnet-Tschudi F (2000). Use of aggregating brain cell cultures to study developmental effects of organophosphorus insecticides. Neurotoxicology.

